# In Memoriam—I. Tisdale Talbot, M. D.

**Published:** 1899-08

**Authors:** 


### IN MEMORIAM—I. TISDALE TALBOT, M. D.

We, the members of the Consulting Board of the Westboro Insane Hospital, shocked and profoundly saddened by the sudden loss of our honored Chairman, Dr. I. Tisdale Talbot, desire to express our grief and our sense of personal bereavement in the sundering of the close ties which have so long united us as men, as physicians and as co-workers upon this Board; as well as our keen realization of the loss to this institution of his wise counsels, his ever-active interest and his ripened experience.

We desire, also, to tender to her who labored with him for the welfare of this hospital, as in many other fields of usefulness, and to the other members of his family, our sincere and heartfelt sympathy.

Howard P. Bellows,
Chas. L. Nichols,
John Prentice Rand,
*For the Board.*

A Fine Opening for the Young Physician.—We are too crowded in the United States, but there is a land where room is plentiful and the country overrun with patients. Russia has a population of 127,000,000 and only 18,334 physicians, or one doctor to every 6,926 inhabitants, while the United States, with a population of 75,000,000, has 120,000 physicians, or one doctor to every 625 inhabitants.—*Charlotte Medical Journal.*

